# Comparison of the Sex Pheromone Composition of *Harmonia axyridis* Originating from Native and Invaded Areas

**DOI:** 10.3390/insects10100326

**Published:** 2019-09-30

**Authors:** Pauline Legrand, Maryse Vanderplanck, Francois J. Verheggen

**Affiliations:** 1 Chemical and Behavioural Ecology, Gembloux Agro-Bio Tech, TERRA, University of Liege, 5030 Gembloux, Belgium; pauline.legrand07@gmail.com; 2 Analytical Chemistry, Gembloux Agro-Bio Tech, University of Liege, 5030 Gembloux, Belgium; Maryse.VANDERPLANCK@umons.ac.be

**Keywords:** multicolored Asian lady beetle, pheromone, sexual communication, invasive species

## Abstract

The multicolored Asian lady beetle, *Harmonia axyridis* (Coleoptera: Coccinellidae), originates from South-East Asia and is now considered as an invasive species at a worldwide scale, with populations encountered in North and South America, Africa, and Europe. Several previous studies suggested that invasive populations display different behavioral and physiological traits, leading to a better fitness than native individuals. *H. axyridis* sex pheromone was identified recently, but only from individuals established in Europe. In this study, we compare the composition of the female sex pheromone of *H. axyridis* from two populations: (i) an invasive population in North America, and (ii) a native population in South-East China. We found the females originating from both populations to release in similar proportions the same five pheromonal compounds, namely β-caryophyllene, β-elemene, methyl-eugenol, α-humulene, and α-bulnesene. However, females from the North American strain release all five compounds in larger amount than the Chinese ones. Whether invasive individuals were selected during the process of invasion through their capacity to better call and find sexual partners remains to be confirmed.

## 1. Introduction

*Harmonia axyridis* Pallas (Coleoptera: Coccinellidae) was introduced to North America in the early 20th century as a biological agent to control hemipteran pests [1]. Seven decades later, the first invasive populations were observed on the eastern and western coasts [2]. Genetic analyses then confirmed that North American individuals spread to South America and Europe in 2001, and to Africa in 2004 [2,3]. Invasive populations are now considered important pests of small fruits and are a nuisance in houses, where they aggregate to overwinter [4,5]. They are also considered a threat to biodiversity, since they feed occasionally on native beneficial insect species [6,7].

Understanding how a new introduced species can perform so well within a very short period of time, even surpassing native species, is of paramount importance [8]. The key to the success of *H. axyridis* is probably an advantageous combination of its ability to complete two to three generations per year, to reproduce in many different niches, and to feed on a wide range of food sources (e.g., [9]). Moreover, recent works also suggest that *H. axyridis* is exhibiting a rapid evolution of various life history traits during its process of invasion, which may include reproductive behavior [10,11,12,13,14].

There is evidence that mate recognition in *H. axyridis* relies on a sex pheromone released by females to attract males before mating [15]. The pheromone blend is composed of one major (β-caryophyllene) and four minor chemical constituents (β-elemene, methyl-eugenol, α-humulene, α-bulnesene). Sex pheromones enhance the ability of a species to reproduce, especially in the case of insect species exhibiting low densities of individuals in recently invaded areas. In this study, we test the hypothesis that the composition of the volatile sex pheromone of native and invasive lady beetles differ quantitatively and qualitatively.

## 2. Methods and Materials

Several hundred *Harmonia axyridis* were captured at different locations in Tai’an (Shandong, China) and in Brookings (South Dakota, USA) and were transferred to the laboratory (Belgium) as adults and kept under the same rearing conditions as described by Fassotte et al. (2014) [15]. To induce oviposition, pea aphids (*Acyrthosiphon pisum*) were introduced in the rearing boxes. Larvae were fed ad libitum with pea aphids until pupation.

We followed the same sex pheromone sampling procedure as described in Fassotte et al. (2014) [15]. Sex pheromone collection was performed on 28–31-day-old virgin adults. Fifteen virgin females were maintained together for 15 days in a 4-L glass chamber (inner diameter: 12 cm; height: 36 cm) in the presence of a *Vicia faba* plant. The insects were fed with sugar lumps during the first five days (from D1 to D5). On the sixth day (D6), sugar was replaced by 3 g of pea aphids. Then, every two days, the same quantity of pea aphids was introduced. Indeed, aphids trigger sexual maturation in *H. axyridis*, which lay eggs in habitats rich in prey [16]. During the entire experiment (from D1 to D15), an airstream was blown into the glass chamber at a flow rate of 700 mL/min using a digital pump. The inflow was purified by passing through an activated charcoal filter, whereas the outflow containing pheromonal compounds was pulled through an adsorbent cartridge containing 30 mg of Haysep Q, a copolymer of ethylvinylbenzene and divinylbenzene (80–100 mesh). A second security cartridge containing 60 mg of the same adsorbent was also added to detect any breakthrough. The volatiles were collected for 24 h before the adsorbent cartridge was changed. After collection, 200 mL of *n*-hexane (purity >95%) were used to elute the trapped volatiles. Each elution sample was capped into a chromatography vial after the addition of 1 μL of *n*-butylbenzene, used as internal standard at 9 μg/μL. The experiment was conducted in a laboratory that was free from any semiochemical use, at 22 ± 2 °C and 50 ± 10 % relative humidity. Headspace sampling in a control glass chamber (i.e., without lady beetles) was also performed to verify the origin of volatile compounds.

To compare qualitatively and quantitatively the chemical profiles, eluted samples were analyzed by coupled gas chromatography/mass spectrometry (Trace GC/MS, ThermoFisher Scientific, Louvain-La-Neuve, Belgium). The GC system was equipped with a HP-5 column (30 m × 0.25 mm × 0.25 µm) coupled with a mass spectrometer (MS). The injector was at 240 °C and the injections were made in splitless mode with 1 μL of each sample. The carrier gas was helium (1.0 mL/min). The initial temperature was held at 40 °C for 2 min followed by a gradual increase of 4 °C/min to 95 °C, then of 6 °C/min to 155 °C, and finally of 25 °C/min to 280 °C. This final temperature was held for 5 min. The mass spectra were recorded in the electron impact mode at 70 eV (source temperature at 200 °C, scanned mass range: 39 to 300 m/z). The detected peaks were identified from their retention data and by comparing the obtained mass spectra with those of pure standards (all purchased at Sigma-Aldrich, Saint-Quentin Fallavier, France). To examine the quantities of each compound emitted by lady beetles, daily collected volatile samples were analyzed by GC (from D1 to D15). The non-polar capillary column (5% phenyl; 30 m × 0.25 mm × 0.25 µm) was combined with a flame ionization detector (FID at 260 °C). One microliter of each extract was injected in splitless mode at 250 °C. The carrier gas was helium (1 mL/min). The temperature was set at 40 °C for 2 min, was then increased to 300 °C at 10 °C/min, and held at 300 °C for 5 min.

We used an ANCOVA (analysis of covariance) to detect the effect of population type (i.e., China or USA) on the absolute (ng) and relative (% of total pheromones) quantities of each compound. This ANCOVA approach took into account the time as covariate in order to analyze whether the pheromone emission of both populations showed the same release dynamics during the experiment. The normality of our data was verified by a Shapiro–Wilk test applied on residuals. Five volatile collections were performed over 15 days for each population. Statistical analyses were conducted using SAS (SAS France, Grégy-sur-Yerres, France) version 9.4.

## 3. Results and Discussion

**Figure 1:**
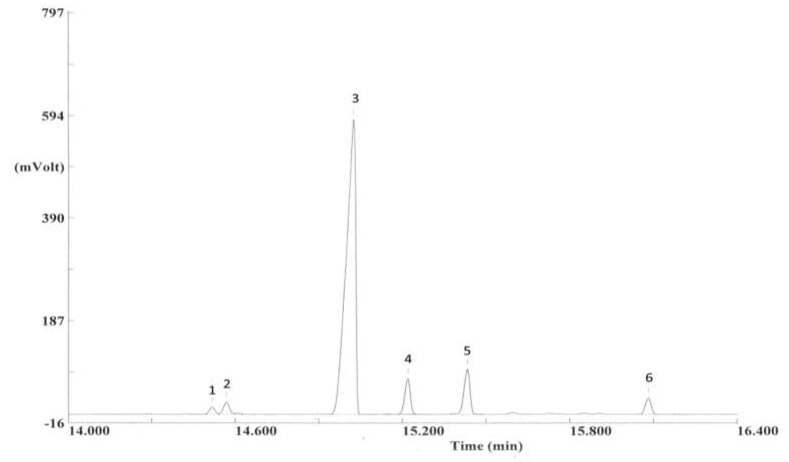
Gas chromatogram of the sex pheromone components emitted by *H. axyridis* females on the 15th day of the experiment. β-Elemene (1); Methyl-eugenol (2); β-Caryophyllene (3); (E)-β-Farnesene (4); α-Humulene (5); α-Bulnesene (6). The internal standard was not injected in this chromatogram. (E)-β-Farnesene (4) is emitted by aphids as an alarm signal [20].

**Figure 2:**
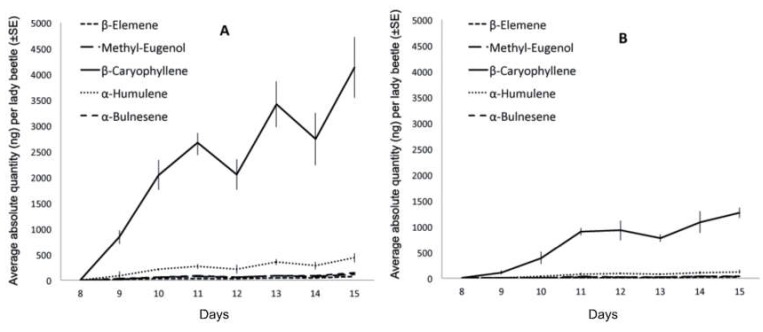
Pheromone emission between the eighth and fifteenth day of the experiment in *H. axyridis* females originating from (**A**) Brookings (USA) and (**B**) Tai’an (China). Lady beetles released no volatile compound before day 8.

Starting on day nine (D9), *H. axyridis* females from both tested populations started to lower their head and thorax, while raising, extending, and contracting their abdomen. Meanwhile, they raised their elytra above their bodies, exposing some of their tergites. These movements were generally repeated several times, and were previously found to be associated with the emission of a sex pheromone. Here, we found that females from both populations released the same five pheromonal compounds found previously by Fassotte et al. (2016) [17] in European populations, namely β-caryophyllene, β-elemene, methyl-eugenol, α-humulene, and α-bulnesene (Figure 1). β-Caryophyllene was previously mentioned as a major volatile of *H. axyridis*, suggesting its implication in pheromonal communication [18,19]. At present, we do not know where these terpenoid compounds are synthesized. In addition, (E)-β-farnesene was detected. Because this molecule was collected from our control chambers (containing plants and aphids only), and because it is commonly considered an aphid pheromone, it was not considered to be a *H. axyridis* volatile [20]. Females from Brookings (USA) emitted concentrations three times higher than females originating from Tai’an (China), both in terms of total blend and individual compounds (*p* < 0.001; Table 1). With regards to the relative abundances, females from Brookings emitted higher proportions of β-elemene (*p* = 0.001) and α-bulnesene (*p* = 0.036), whereas females originating from Tai’an emitted a higher proportion of methyl-eugenol (*p* = 0.001) (Table 1). Over the seven days of pheromone emission (from D9 to D15), methyl-eugenol was the only compound displaying differing release dynamics between the populations in terms of absolute (*p* = 0.048) and relative (*p* < 0.001) quantities (Table 1). Specifically, methyl-eugenol showed an uneven evolution in females from Brookings populations, whereas it displayed a quite constant increase over time in females from Tai’an populations (Figure 2).

Since beetles from the North American populations produce a larger amount of sex pheromone, we speculate that they are able to attract male partners from longer distances than native populations. Such an adaptation would be a tremendous asset to increase fitness and colonize new areas where conspecifics are sparse. Also, several studies have suggested that the invasiveness of *H. axyridis* would be achieved through evolutionary shifting in reproductive strategy and associated traits during the invasion process [3,10,11,12]. With such high concentrations of volatile emissions, it is not surprising to observe variation in the relative proportions of some volatile constituents. Whether these constituents increase the behavioral activity of *H. axyridis* sex pheromone remains uncertain.

Based on these results, we surmise that the low density of individuals observed during the early invasions (i.e., in North America) leads to the selection of females releasing higher amounts of pheromone, which results in a more effective attraction of sexual partners [11]. Our results shed new light on the selective pressures that might have contributed to the invasiveness of *H. axyridis*. Further studies should be completed on additional populations from various invaded areas in order to substantiate our findings.

## Figures and Tables

**Table 1:** ANCOVA tables performed on absolute and relative abundances. *F* statistics and *p* values are mentioned for population and population × time effects.

Sex Pheromone Components	ANCOVA Table (Absolute Abundance)				ANCOVA Table (Relative Proportions)			
	Population		Population × Time		Population		Population × Time	
	*F*	*p*	*F*	*p*	*F*	*p*	*F*	*p*
β-Elemene	32.630	<0.001	3.610	0.067	13.22	0.001	0.400	0.534
Methyl-eugenol	30.460	<0.001	4.260	0.048	12.60	0.001	20.99	<0.001
β-Caryophyllene	28.040	<0.001	2.360	0.135	0.300	0.588	1.740	0.198
α-Humulene	31.540	<0.001	2.880	0.100	0.330	0.567	0.640	0.432
α-Bulnesene	28.390	<0.001	3.000	0.094	4.810	0.036	2.100	0.158
Total	28.600	<0.001	2.480	0.126				

## References

[1] Gordon R.D. (1985). The Coleoptera (Coccinellidae) of America north of Mexico. J. N. Y. Entomol. Soc..

[2] Brown P.M.J., Thomas C.E., Lombaert E., Jeffries D.L., Estoup A., Lawson Handley L.J. (2011). The Global Spread of Harmonia Axyridis (Coleoptera: Coccinellidae): Distribution, Dispersal and Routes of Invasion. Biocontrol.

[3] Lombaert E., Guillemaud T., Cornuet J.M., Malausa T., Facon B., Estoup A. (2010). Bridgehead Effect in the Worldwide Invasion of the Biocontrol Harlequin Ladybird. PLoS ONE.

[4] Koch R.L. (2003). The multicolored Asian lady beetle, *Harmonia axyridis*: A review of its biology, uses in biological control, and non-target impacts. J. Insect Sci..

[5] Durieux D., Fischer C., Brostaux Y., Sloggett J.J., Deneubourg J.-L., Vandereycken A., Joie E., Wathelet J.-P., Lognay G., Haubruge E. (2012). Role of long-chain hydrocarbons in the aggregation behaviour of *Harmonia axyridis* (Pallas) (Coleoptera: Coccinellidae). J. Insect Physiol..

[6] Ingels B., Aebi A., Hautier L., Van Leeuwen T., De Clercq P. (2013). Molecular analysis of the gut contents of harmonia axyridis (coleoptera: Coccinellidae) as a method for detecting intra-guild predation by this species on aphidophagous predators other than coccinellids. Eur. J. Entomol..

[7] Vandereycken A., Durieux D., Joie E., Sloggett J.J., Haubruge E., Verheggen F.J. (2013). Is the multicolored asian ladybeetle, *Harmonia axyridis*, the most abundant natural enemy to aphids in agroecosystems?. J. Insect Sci..

[8] Verheggen F.J., Vogel H., Vilcinskas A. (2017). Behavioral and immunological features promoting the invasive performance of the harlequin ladybird *Harmonia axyridis*. Front. Ecol. Evol..

[9] Vandereycken A., Durieux D., Joie E., Haubruge E., Verheggen F.J. (2012). Habitat diversity of the multicolored asian ladybeetle *Harmonia axyridis* pallas (Coleoptera: Coccinellidae) in agricultural and arboreal ecosystems: A review. Biotechnol. Agron. Soc. Environ..

[10] Facon B., Hufbauer R.A., Tayeh A., Loiseau A., Lombaert E., Vitalis R., Guillemaud T., Lundgren J.G., Estoup A. (2011). Inbreeding Depression Is Purged in the Invasive Insect *Harmonia axyridis*. Curr. Biol..

[11] Laugier G.J.M., Le Moguédec G., Tayeh A., Loiseau A., Osawa N., Estoup A., Facon B. (2013). Increase in Male Reproductive Success and Female Reproductive Investment in Invasive Populations of the Harlequin Ladybird Harmonia Axyridis. PLoS ONE.

[12] Tayeh A., Estoup A., Lombaert E., Guillemaud T., Kirichenko N., Lawson-Handley L., De Clercq P., Facon B. (2014). Cannibalism in Invasive, Native and Biocontrol Populations of the Harlequin Ladybird. BMC Evol. Biol..

[13] Tayeh A., Hufbauer R.A., Estoup A., Ravigné V., Frachon L., Facon B. (2015). Biological invasion and biological control select for different life histories. Nat. Commun..

[14] Wang X., Yang X., Zang L., Wang Z., Ruan C., Liu X. (2017). Effect of geographic variation on biology and cold tolerance of *Harmonia axyridis* in China. Entomol. Gen..

[15] Fassotte B., Fischer C., Durieux D., Lognay G., Haubruge E., Francis F., Verheggen F.J. (2014). First Evidence of a Volatile Sex Pheromone in Lady Beetles. PLoS ONE.

[16] Obata S. (1997). The Influence of Aphids on the Behaviour of Adults of the Ladybird Beetle, *Harmonia axyridis* (Col.: Coccinellidae). Entomophaga.

[17] Fassotte B., Francis F., Verheggen F.J. (2016). The Scent of Love: How Important Are Semiochemicals in the Sexual Behavior of Lady Beetles?. J. Pest Sci..

[18] Brown A.E., Riddick E.W., Aldrich J.R., Holmes W.E. (2006). Identification of (-) B-caryophyllene as a gender specific terpene produced by the multicolored Asian lady beetle. J. Chem. Ecol..

[19] Verheggen F.J., Fagel Q., Heuskin S., Lognay G., Francis F., Haubruge E. (2007). Electrophysiological and behavioral responses of the multicolored asian lady beetle, *Harmonia axyridis* Pallas, to sesquiterpene semiochemicals. J. Chem. Ecol..

[20] Vandermoten S., Mescher M.C., Francis F., Haubruge E., Verheggen F.J. (2012). Aphid alarm pheromone: An overview of current knowledge on biosynthesis and functions. Insect Biochem. Mol. Biol..

